# Complete Genome Sequence of *Bacillus subtilis* BAB-1, a Biocontrol Agent for Suppression of Tomato Gray Mold

**DOI:** 10.1128/genomeA.00744-14

**Published:** 2014-08-07

**Authors:** Qinggang Guo, Shezeng Li, Xiuyun Lu, Xiaoyun Zhang, Peipei Wang, Ping Ma

**Affiliations:** Institute of Plant Protection, Hebei Academy of Agricultural and Forestry Sciences, Integrated Pest Management Center of Hebei Province, and Key Laboratory of IPM on Crops in Northern Region of North China, Ministry of Agriculture, Baoding, China

## Abstract

*Bacillus subtilis* BAB-1, isolated from cotton rhizosphere soil, is an excellent biocontrol agent for tomato gray mold. The genome of *B. subtilis* strain BAB-1 was fully sequenced and annotated, genes encoding the antifungal active compound were identified, and multiple sets of regulatory systems were found in the genome.

## GENOME ANNOUNCEMENT

*Bacillus subtilis* has been widely studied and used for plant disease control. The contributing factors to the success of this bacterium include the production of various antimicrobial compounds (1), competition for nutrition and niche with plant pathogens (2), and induced systemic resistance to pathogens (3). *B. subtilis* strain BAB-1, isolated from cotton rhizosphere soil, showed strong inhibitory ability against the growth of phytopathogens *in vitro*. In addition, strain BAB-1 had been identified as an excellent biocontrol agent for tomato gray mold (4). Phylogenetic trees based on the *gyrB* and *phoR* gene sequences revealed that strain BAB-1 was closely related to *B. subtilis* 168 (5). To clarify the biocontrol mechanism and facilitate the detection of strain BAB-1, we sequenced and annotated the complete genome sequence of the strain.

Whole-genome sequencing of strain BAB-1 was performed with an Illumina GA IIx analyzer at the Beijing Genomics Institute (BGI) in China. Sequence reads were generated from a 500-bp paired-end library, giving 600-Mb sequences with approximately 120× genome sequence coverage. The paired reads were *de novo* assembled with SOAPdenovo v. 1.03 software (BGI) (http://soap.genomics.org.cn/soapdenovo.html) based on the genome sequence of type strain *B. subtilis* 168 (AL009126.3), and 98.6% of the reads were assembled into 23 large scaffolds. Most of the gaps within the scaffolds were filled by local assembly of Solexa reads. The remaining gaps between scaffolds were filled by sequencing of PCR amplification using an ABI 3730 sequencer. Protein-encoding genes, rRNA operons, and tRNAs were predicted by Glimmer v. 3.0 (6). Annotation was performed using the Rapid Annotation Using Subsystem Technology (RAST) server (7).

The complete genome sequence of strain BAB-1 is composed of a circular 4,021,944-bp chromosome with mean GC content of 43.89%. There are 4,104 coding genes, 376 rRNAs, and 48 tRNAs in the chromosome. Approximately 5.2% of the strain BAB-1 genome is devoted to synthesis of antimicrobial products, including nonribosomal peptide synthetase (NRPS) antibiotics, polyketide synthase (PKS) antibiotics, and lantibiotics, as well as bacillibactin. Among those products, the lipopeptides surfactin and fengycin were identified from strain BAB-1 by matrix-assisted laser desorption ionization–time of flight mass spectrometry (MALDI-TOF MS). The fengycin was identified as a major antifungal active compound in inhibition against the growth of *Botrytis cinerea*, and when applied in combination with fengycin, the surfactin showed synergistic actions, which were confirmed by antifungal assay *in vivo* (8). The iturin lipopeptides have a strong antifungal ability, but gene clusters for iturin lipopeptides are absent from the genome of strain BAB-1. As in other prokaryotes, two-component signal transduction systems of *B. subtilis* are important elements of the adaptive response to a variety of external conditions (9). A total of 35 two-component systems (TCS) were identified from the genome of strain BAB-1, including the biocontrol function-related TCS *comP*/*comA* (10), *phoR*/*phoP* (11), and *degU*/*degS* (12). Finally, we identified 86 transposons, 25 phage-like elements, and type III restriction modification systems in strain BAB-1 genome.

### Nucleotide sequence accession number.

The complete genome sequence has been deposited in GenBank under accession no. CP004405.

## References

[1] Stein T 2005 *Bacillus subtilis* antibiotics: structures, syntheses and specific functions. Mol. Microbiol. 56:845–857. 10.1111/j.1365-2958.2005.04587.x 15853875

[2] Morris CE Monier JM 2003 The ecological significance of biofilm formation by plant-associated bacteria. Annu. Rev. Phytopathol. 41:429–453. 10.1146/annurev.phyto.41.022103.134521 12730399

[3] Desoignies N Schramme F Ongena M Legrève A 2013 Systemic resistance induced by *Bacillus* lipopeptides in *Beta vulgaris* reduces infection by the rhizomania disease vector *Polymyxa betae*. Mol. Plant Pathol. 14:416–421. 10.1111/mpp.12008 23279057 PMC6638685

[4] Wang WQ Ma P Han XY Lu XY Zhang XF 2011 Control effect of joint application of bio-fungicides and synthesized fungicides on cucumber diseases. Chin. J. Biol. Control 1:104–109

[5] Guo Q Li S Lu X Li B Stummer B Dong W Ma P 2012 *phoR* sequences as a phylogenetic marker to differentiate the species in the *Bacillus subtilis* group. Can. J. Microbiol. 58:1295–1305. 10.1139/w2012-106 23145827

[6] Delcher AL Bratke KA Powers EC Salzberg SL 2007 Identifying bacterial genes and endosymbiont DNA with Glimmer. Bioinformatics 23:673–679. 10.1093/bioinformatics/btm009 17237039 PMC2387122

[7] Aziz RK Bartels D Best AA DeJongh M Disz T Edwards RA Formsma K Gerdes S Glass EM Kubal M Meyer F Olsen GJ Olson R Osterman AL Overbeek RA McNeil LK Paarmann D Paczian T Parrello B Pusch GD Reich C Stevens R Vassieva O Vonstein V Wilke A Zagnitko O 2008 The RAST server: rapid annotations using subsystems technology. BMC Genomics 9:75. 10.1186/1471-2164-9-75 18261238 PMC2265698

[8] Li B Lu X Guo Q Qian C Li S Ma P 2010 Isolation and identification of lipopeptides and volatile compounds produced by *Bacillus subtilis* strain BAB-1. Sci. Agric. Sinica 43:3547–3554

[9] Yang SJ Bayer AS Mishra NN Meehl M Ledala N Yeaman MR Xiong YQ Cheung AL 2012 The *Staphylococcus aureus* two-component regulatory system, GraRS, senses and confers resistance to selected cationic antimicrobial peptides. Infect. Immun. 80:74–81. 10.1128/IAI.05669-11 21986630 PMC3255649

[10] Wang X Luo C Liu Y Nie Y Liu Y Zhang R Chen Z 2010 Three nonaspartate amino acid mutations in the ComA response regulator receiver motif severely decrease surfactin production, competence development and spore formation in *Bacillus subtilis*. J. Microbiol. Biotechnol. 20:301–310. 10.1016/j.nbt.2009.06.975 20208433

[11] Guo Q Li S Lu X Li B Ma P 2010 PhoR/PhoP two-component regulatory system affects biocontrol capability of *Bacillus subtilis* NCD-2. Genet. Mol. Biol. 33:333–340. 10.1590/S1415-47572010005000032 21637491 PMC3036859

[12] Mariappan A Makarewicz O Chen XH Borriss R 2012 Two-component response regulator DegU controls the expression of bacilysin in plant-growth-promoting bacterium *Bacillus amyloliquefaciens* FZB42. J. Mol. Microbiol. Biotechnol. 22:114–125. 10.1159/000338804 22677979

